# *trans*-Carbonyl­chloridobis[diphen­yl(4-vinyl­phen­yl)phosphane-κ*P*]rhodium(I)

**DOI:** 10.1107/S1600536812013669

**Published:** 2012-04-04

**Authors:** Hezron Ogutu, Leo Kirsten, Reinout Meijboom

**Affiliations:** a Research Centre for Synthesis and Catalysis, Department of Chemistry, University of Johannesburg, PO Box 524, Auckland Park, Johannesburg 2006, South Africa

## Abstract

In the title compound, *trans*-[RhCl(C_20_H_17_P)_2_(CO)], the Rh^I^ atom is situated on a center of symmetry, resulting in a statistical 1:1 disorder of the chloride [Rh—Cl = 2.383 (2) Å] and carbonyl [Rh—C = 1.752 (7) Å] ligands. The distorted *trans* square-planar environment is completed by two P atoms [Rh—P = 2.3251 (4) Å] from two diphen­yl(4-vinyl­phen­yl)phosphane ligands. The vinyl group is disordered over two sets of sites in a 0.668 (10):0.332 (10) ratio. The crystal packing exhibits weak C—H⋯Cl and C—H⋯O hydrogen bonds and π–π inter­actions between the phenyl rings of neighbouring mol­ecules, with a centroid–centroid distance of 3.682 (2) Å.

## Related literature

For a review of rhodium Vaska {*trans*-[RhCl(CO)(P*R*_3_)_2_]} compounds, see: Roodt *et al.* (2003 ▶). For related compounds, see: Angoletta (1959 ▶); Vaska & Di Luzio (1961 ▶); Chen *et al.* (1991 ▶); Kuwabara & Bau (1994 ▶); Otto *et al.* (2000 ▶); Otto (2001 ▶); Meijboom *et al.* (2005 ▶). 
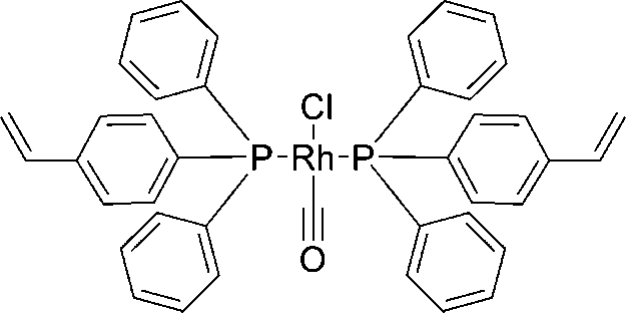


## Experimental

### 

#### Crystal data

[RhCl(C_20_H_17_P)_2_(CO)]
*M* *_r_* = 742.98
Triclinic, 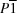

*a* = 9.9030 (4) Å
*b* = 9.9310 (4) Å
*c* = 10.4150 (4) Å
α = 85.727 (2)°
β = 68.475 (2)°
γ = 62.295 (2)°
*V* = 837.85 (6) Å^3^
*Z* = 1
Cu *K*α radiation
μ = 6.01 mm^−1^
*T* = 100 K
0.10 × 0.08 × 0.06 mm

#### Data collection

Bruker APEXII CCD diffractometer
Absorption correction: multi-scan (*SADABS*; Bruker, 2007 ▶) *T*_min_ = 0.107, *T*_max_ = 0.402
11163 measured reflections
2941 independent reflections
2850 reflections with *I* > 2σ(*I*)
*R*_int_ = 0.026

#### Refinement

*R*[*F* ^2^ > 2σ(*F* ^2^)] = 0.024
*wR*(*F* ^2^) = 0.057
*S* = 1.04
2941 reflections
232 parameters
6 restraints
H-atom parameters constrained
Δρ_max_ = 0.46 e Å^−3^
Δρ_min_ = −0.27 e Å^−3^

### 

Data collection: *APEX2* (Bruker, 2007 ▶); cell refinement: *SAINT-Plus* (Bruker, 2007 ▶); data reduction: *SAINT-Plus*; program(s) used to solve structure: *SHELXS97* (Sheldrick, 2008 ▶); program(s) used to refine structure: *SHELXL97* (Sheldrick, 2008 ▶); molecular graphics: *DIAMOND* (Brandenburg & Putz, 2005 ▶); software used to prepare material for publication: *WinGX* (Farrugia, 1999 ▶).

## Supplementary Material

Crystal structure: contains datablock(s) global, I. DOI: 10.1107/S1600536812013669/cv5270sup1.cif

Structure factors: contains datablock(s) I. DOI: 10.1107/S1600536812013669/cv5270Isup2.hkl

Additional supplementary materials:  crystallographic information; 3D view; checkCIF report

## Figures and Tables

**Table 1:** Hydrogen-bond geometry (Å, °)

*D*—H⋯*A*	*D*—H	H⋯*A*	*D*⋯*A*	*D*—H⋯*A*
C9*B*—H9*B*1⋯O01^i^	0.93	2.54	3.205 (11)	129
C14—H14⋯Cl1^ii^	0.93	2.79	3.660 (3)	157

Symmetry codes: (i) 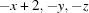; (ii) 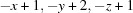.

**Fig. 1.**
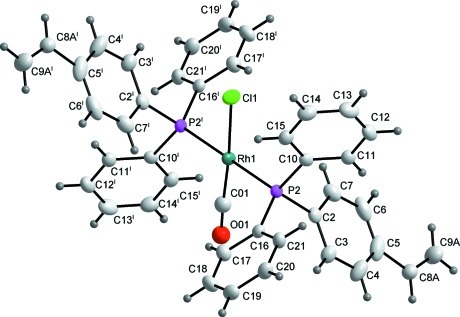
The molecular structure of the title compound showing the atomic numbering and 50% probability displacement ellipsoids [symmetry code: (i) (1 - x, 1 - y, 1 - z)].

## supplementary crystallographic information

### Comment

The original Vaska complex, trans-[IrCl(CO)(PPh_3_)_2_], was first reported in 1959 (Angoletta, 1959), but later correctly formulated by Vaska in 1961 (Vaska & Di Luzio, 1961) . This class of symmetrical square-planar complexes often crystallizes with the metal atom on a crystallographic inversion centre of symmetry, thus imposing a disordered packing arrangement (Otto, 2001;Otto et al., 2000; Chen et al.,1991; Kuwabara & Bau, 1994).These Vaska type complexes are useful model complexes and provide several probing methods, e.g. NMR and IR, to investigate the steric and electronic effects of novel group 15 ligands (Roodt et al., 2003).

Here we report the title compound, the i>trans-[RhClL_2_(CO)](L = diphenyl(4-vinylphenyl)phosphane) complex crystallizes in the triclinic space group, P-1.The crystal structure of the title compound (Fig.1) shows the expected square planar geometry with the phosphane ligands trans to each other. The Rh^I^ atom is situated on a center of symmetry, resulting in a statistical 1:1 disorder of the chlorido [Rh—Cl 2.383 (2) Å] and carbonyl [Rh—C 1.752 (7) Å] ligands. The distorted trans square-planar environment is completed by two P atoms [Rh—P 2.3251 (4) Å] from two L ligands. The vinyl group is disordered over two sets of sites in a 0.668 (10):0.332 (10) ratio. The J coupling of (Rh-P) is 128 Hz which is in agreement with the coupling constants for other rhodium Vaska type complexes of this nature (Meijboom et al., 2005). The C01–Rh1–P2 angle of 92.99 (17) ° and the P2–Rh1–Cl1 of 94.46 (3) ° exemplifies the deviation from the ideal 90 ° square planar geometry.

The crystal packing exhibits weak intermolecular C—H···Cl and C—H···O hydrogen bonds (Table 1). There is a π–π interaction between the neighbouring phenyl ring centroids of C16-C21 and C16-C21 (2-x,1-y,1-z), respectively with the centroid-centroid distance of 3.682 (2) Å.

### Experimental

Diphenylphosphinostyrene (0.15 g, 0.51 mmol) was dissolved in acetone (6 cm^3^). A solution of dichlorotetracarbonyldirhodium(I) (0.04 g, 0.13 mmol) in acetone was added to the phosphine solution. The mixture was stirred for 5 minutes, slow evaporation of the solvent afforded the title compound as a yellow crystalline solid. Spectroscopic analysis:^31^P{H} NMR (CDCl_3_, 161.99 MHz, p.p.m.): 46.42 [d, ^1^J(Rh—P) = 179.81 Hz]; IR ν(CO): 1957.96 cm^-1^; (CD_2_Cl_2_) ν(CO): 1977.04 cm^-1^.

### Refinement

The H atoms were placed in geometrically idealized positions (C—H bonds of 0.95–0.98 /%A) and constrained to ride on their parent atoms with *U*_iso_(H) = 1.2*U*_eq_(C).

### Crystal data

**Table:**

[RhCl(C_20_H_17_P)_2_(CO)]	*Z* = 1
*M**_r_* = 742.98	*F*(000) = 380
Triclinic, *P*1	*D*_x_ = 1.473 Mg m^−^^3^
Hall symbol: -P 1	Cu *K*α radiation, λ = 1.54178 Å
*a* = 9.9030 (4) Å	Cell parameters from 8410 reflections
*b* = 9.9310 (4) Å	θ = 4.6–66.3°
*c* = 10.4150 (4) Å	µ = 6.01 mm^−^^1^
α = 85.727 (2)°	*T* = 100 K
β = 68.475 (2)°	Rectangular, yellow
γ = 62.295 (2)°	0.10 × 0.08 × 0.06 mm
*V* = 837.85 (6) Å^3^	

### Data collection

**Table:**

Bruker APEXII CCD diffractometer	2941 independent reflections
Radiation source: fine-focus sealed tube	2850 reflections with *I* > 2σ(*I*)
Graphite monochromator	*R*_int_ = 0.026
φ and ω scans	θ_max_ = 66.3°, θ_min_ = 4.6°
Absorption correction: multi-scan (*SADABS*; Bruker, 2007)	*h* = −11→7
*T*_min_ = 0.107, *T*_max_ = 0.402	*k* = −11→11
11163 measured reflections	*l* = −12→12

### Refinement

**Table:**

Refinement on *F*^2^	Primary atom site location: structure-invariant direct methods
Least-squares matrix: full	Secondary atom site location: difference Fourier map
*R*[*F*^2^ > 2σ(*F*^2^)] = 0.024	Hydrogen site location: inferred from neighbouring sites
*wR*(*F*^2^) = 0.057	H-atom parameters constrained
*S* = 1.04	*w* = 1/[σ^2^(*F*_o_^2^) + (0.0256*P*)^2^ + 0.5512*P*] where *P* = (*F*_o_^2^ + 2*F*_c_^2^)/3
2941 reflections	(Δ/σ)_max_ = 0.001
232 parameters	Δρ_max_ = 0.46 e Å^−^^3^
6 restraints	Δρ_min_ = −0.27 e Å^−^^3^

### Special details

**Table:**

Geometry. All e.s.d.'s (except the e.s.d. in the dihedral angle between two l.s. planes) are estimated using the full covariance matrix. The cell e.s.d.'s are taken into account individually in the estimation of e.s.d.'s in distances, angles and torsion angles; correlations between e.s.d.'s in cell parameters are only used when they are defined by crystal symmetry. An approximate (isotropic) treatment of cell e.s.d.'s is used for estimating e.s.d.'s involving l.s. planes.
Refinement. Refinement of *F*^2^ against ALL reflections. The weighted *R*-factor *wR* and goodness of fit *S* are based on *F*^2^, conventional *R*-factors *R* are based on *F*, with *F* set to zero for negative *F*^2^. The threshold expression of *F*^2^ > σ(*F*^2^) is used only for calculating *R*-factors(gt) *etc*. and is not relevant to the choice of reflections for refinement. *R*-factors based on *F*^2^ are statistically about twice as large as those based on *F*, and *R*- factors based on ALL data will be even larger.

### Fractional atomic coordinates and isotropic or equivalent isotropic displacement parameters (Å^2^)

**Table:**

	*x*	*y*	*z*	*U*_iso_*/*U*_eq_	Occ. (<1)
Rh1	0.5	0.5	0.5	0.02293 (9)	
Cl1	0.34866 (19)	0.7717 (2)	0.55041 (12)	0.0329 (3)	0.5
C01	0.6149 (7)	0.2999 (8)	0.4747 (5)	0.0330 (14)*	0.5
O01	0.6932 (5)	0.1680 (6)	0.4511 (4)	0.0393 (13)*	0.5
P2	0.71286 (5)	0.50159 (5)	0.30681 (4)	0.02055 (12)	
C2	0.7589 (2)	0.3785 (2)	0.15917 (19)	0.0239 (4)	
C3	0.9177 (3)	0.2746 (2)	0.0745 (2)	0.0305 (4)	
H3	1.0063	0.2629	0.0949	0.037*	
C4	0.9461 (3)	0.1880 (3)	−0.0400 (2)	0.0412 (5)	
H4	1.0536	0.1179	−0.0942	0.049*	
C5	0.8188 (4)	0.2033 (3)	−0.0755 (2)	0.0431 (6)	
C6	0.6589 (3)	0.3064 (3)	0.0108 (3)	0.0421 (6)	
H6	0.5708	0.3183	−0.0103	0.05*	
C7	0.6284 (3)	0.3916 (2)	0.1273 (2)	0.0331 (4)	
H7	0.5206	0.4578	0.1843	0.04*	
C10	0.6760 (2)	0.6851 (2)	0.23862 (19)	0.0224 (4)	
C11	0.6826 (2)	0.7072 (2)	0.1032 (2)	0.0253 (4)	
H11	0.7081	0.6263	0.0436	0.03*	
C12	0.6512 (3)	0.8499 (2)	0.0569 (2)	0.0327 (4)	
H12	0.6555	0.8642	−0.0335	0.039*	
C13	0.6137 (3)	0.9700 (2)	0.1445 (3)	0.0379 (5)	
H13	0.591	1.0657	0.1137	0.045*	
C14	0.6097 (3)	0.9485 (2)	0.2788 (2)	0.0346 (5)	
H14	0.5864	1.0292	0.3374	0.042*	
C15	0.6406 (2)	0.8070 (2)	0.3253 (2)	0.0277 (4)	
H15	0.6377	0.793	0.4153	0.033*	
C16	0.9104 (2)	0.4379 (2)	0.32389 (18)	0.0226 (4)	
C17	0.9552 (3)	0.3347 (2)	0.4169 (2)	0.0297 (4)	
H17	0.8835	0.299	0.4721	0.036*	
C18	1.1066 (3)	0.2846 (2)	0.4277 (2)	0.0335 (4)	
H18	1.1369	0.2139	0.4888	0.04*	
C19	1.2126 (2)	0.3398 (2)	0.3477 (2)	0.0316 (4)	
H19	1.3137	0.3065	0.3553	0.038*	
C20	1.1679 (2)	0.4442 (3)	0.2569 (2)	0.0314 (4)	
H20	1.2384	0.4822	0.2042	0.038*	
C21	1.0179 (2)	0.4927 (2)	0.2438 (2)	0.0274 (4)	
H21	0.9891	0.562	0.1814	0.033*	
C8A	0.8768 (8)	0.0979 (6)	−0.2041 (5)	0.0314 (11)	0.668 (10)
H8A	0.9865	0.0231	−0.2409	0.038*	0.668 (10)
C9A	0.7794 (5)	0.1079 (5)	−0.2647 (4)	0.0441 (14)	0.668 (10)
H9A1	0.6693	0.1819	−0.2295	0.053*	0.668 (10)
H9A2	0.8198	0.041	−0.3433	0.053*	0.668 (10)
C9B	0.9234 (11)	0.0621 (10)	−0.2909 (12)	0.043 (3)	0.332 (10)
H9B1	1.0259	0.0295	−0.2862	0.051*	0.332 (10)
H9B2	0.916	0.0272	−0.3672	0.051*	0.332 (10)
C8B	0.7895 (13)	0.1573 (11)	−0.1898 (8)	0.028 (2)	0.332 (10)
H8B	0.6847	0.1928	−0.1903	0.034*	0.332 (10)

### Atomic displacement parameters (Å^2^)

**Table:**

	*U*^11^	*U*^22^	*U*^33^	*U*^12^	*U*^13^	*U*^23^
Rh1	0.01987 (12)	0.02210 (12)	0.02175 (12)	−0.01035 (8)	−0.00276 (8)	0.00663 (7)
Cl1	0.0272 (6)	0.0269 (8)	0.0345 (6)	−0.0141 (6)	0.0001 (4)	0.0063 (6)
P2	0.0190 (2)	0.0228 (2)	0.0180 (2)	−0.01070 (18)	−0.00469 (17)	0.00582 (17)
C2	0.0292 (9)	0.0246 (9)	0.0233 (9)	−0.0173 (8)	−0.0107 (8)	0.0098 (7)
C3	0.0334 (10)	0.0332 (11)	0.0265 (10)	−0.0203 (9)	−0.0065 (8)	0.0024 (8)
C4	0.0518 (14)	0.0439 (13)	0.0288 (11)	−0.0326 (11)	−0.0013 (10)	−0.0035 (9)
C5	0.0767 (17)	0.0471 (13)	0.0270 (11)	−0.0487 (13)	−0.0171 (11)	0.0118 (10)
C6	0.0690 (16)	0.0469 (13)	0.0488 (13)	−0.0451 (13)	−0.0427 (13)	0.0280 (11)
C7	0.0363 (11)	0.0316 (11)	0.0426 (12)	−0.0208 (9)	−0.0216 (9)	0.0135 (9)
C10	0.0172 (8)	0.0230 (9)	0.0246 (9)	−0.0103 (7)	−0.0051 (7)	0.0061 (7)
C11	0.0225 (9)	0.0273 (9)	0.0248 (9)	−0.0127 (7)	−0.0069 (7)	0.0052 (7)
C12	0.0315 (10)	0.0335 (11)	0.0308 (10)	−0.0154 (9)	−0.0114 (8)	0.0146 (8)
C13	0.0359 (11)	0.0251 (10)	0.0481 (13)	−0.0151 (9)	−0.0119 (10)	0.0133 (9)
C14	0.0317 (10)	0.0264 (10)	0.0405 (12)	−0.0146 (8)	−0.0061 (9)	−0.0009 (8)
C15	0.0247 (9)	0.0302 (10)	0.0249 (9)	−0.0135 (8)	−0.0050 (7)	0.0028 (8)
C16	0.0229 (9)	0.0241 (9)	0.0182 (8)	−0.0094 (7)	−0.0067 (7)	−0.0001 (7)
C17	0.0337 (10)	0.0297 (10)	0.0298 (10)	−0.0168 (8)	−0.0144 (8)	0.0077 (8)
C18	0.0373 (11)	0.0304 (10)	0.0371 (11)	−0.0133 (9)	−0.0230 (9)	0.0086 (9)
C19	0.0273 (10)	0.0353 (11)	0.0315 (10)	−0.0109 (8)	−0.0142 (8)	−0.0023 (8)
C20	0.0274 (10)	0.0418 (12)	0.0266 (10)	−0.0184 (9)	−0.0088 (8)	0.0028 (8)
C21	0.0271 (9)	0.0344 (10)	0.0213 (9)	−0.0149 (8)	−0.0096 (8)	0.0056 (8)
C8A	0.032 (2)	0.030 (2)	0.029 (3)	−0.0122 (19)	−0.010 (2)	0.0010 (17)
C9A	0.044 (2)	0.045 (2)	0.038 (2)	−0.0152 (17)	−0.0148 (16)	−0.0073 (17)
C9B	0.046 (6)	0.048 (5)	0.033 (6)	−0.018 (4)	−0.016 (4)	−0.004 (4)
C8B	0.023 (4)	0.029 (4)	0.031 (4)	−0.010 (4)	−0.011 (3)	−0.002 (3)

### Geometric parameters (Å, º)

**Table:**

Rh1—C01^i^	1.752 (7)	C12—C13	1.377 (3)
Rh1—C01	1.752 (7)	C12—H12	0.93
Rh1—P2	2.3251 (4)	C13—C14	1.388 (3)
Rh1—P2^i^	2.3251 (4)	C13—H13	0.93
Rh1—Cl1^i^	2.383 (2)	C14—C15	1.383 (3)
Rh1—Cl1	2.383 (2)	C14—H14	0.93
C01—O01	1.158 (7)	C15—H15	0.93
P2—C2	1.8205 (19)	C16—C17	1.389 (3)
P2—C10	1.8266 (18)	C16—C21	1.395 (3)
P2—C16	1.8298 (18)	C17—C18	1.389 (3)
C2—C3	1.388 (3)	C17—H17	0.93
C2—C7	1.396 (3)	C18—C19	1.387 (3)
C3—C4	1.387 (3)	C18—H18	0.93
C3—H3	0.93	C19—C20	1.377 (3)
C4—C5	1.380 (4)	C19—H19	0.93
C4—H4	0.93	C20—C21	1.389 (3)
C5—C6	1.396 (4)	C20—H20	0.93
C5—C8B	1.473 (8)	C21—H21	0.93
C5—C8A	1.518 (6)	C8A—C9A	1.299 (8)
C6—C7	1.387 (3)	C8A—H8A	0.93
C6—H6	0.93	C9A—H9A1	0.93
C7—H7	0.93	C9A—H9A2	0.93
C10—C15	1.392 (3)	C9B—C8B	1.311 (15)
C10—C11	1.393 (3)	C9B—H9B1	0.93
C11—C12	1.392 (3)	C9B—H9B2	0.93
C11—H11	0.93	C8B—H8B	0.93
			
C01^i^—Rh1—C01	180.0000 (10)	C12—C11—C10	120.20 (19)
C01^i^—Rh1—P2	92.99 (17)	C12—C11—H11	119.9
C01—Rh1—P2	87.01 (17)	C10—C11—H11	119.9
C01^i^—Rh1—P2^i^	87.01 (17)	C13—C12—C11	120.2 (2)
C01—Rh1—P2^i^	92.99 (17)	C13—C12—H12	119.9
P2—Rh1—P2^i^	180.00 (2)	C11—C12—H12	119.9
C01^i^—Rh1—Cl1^i^	175.46 (17)	C12—C13—C14	120.06 (19)
C01—Rh1—Cl1^i^	4.54 (17)	C12—C13—H13	120
P2—Rh1—Cl1^i^	85.54 (3)	C14—C13—H13	120
P2^i^—Rh1—Cl1^i^	94.46 (3)	C15—C14—C13	119.9 (2)
C01^i^—Rh1—Cl1	4.54 (17)	C15—C14—H14	120.1
C01—Rh1—Cl1	175.46 (17)	C13—C14—H14	120.1
P2—Rh1—Cl1	94.46 (3)	C14—C15—C10	120.72 (19)
P2^i^—Rh1—Cl1	85.54 (3)	C14—C15—H15	119.6
Cl1^i^—Rh1—Cl1	180.00 (6)	C10—C15—H15	119.6
O01—C01—Rh1	176.7 (5)	C17—C16—C21	119.13 (17)
C2—P2—C10	103.30 (8)	C17—C16—P2	120.51 (15)
C2—P2—C16	105.19 (8)	C21—C16—P2	120.36 (14)
C10—P2—C16	102.16 (8)	C16—C17—C18	120.31 (19)
C2—P2—Rh1	110.70 (6)	C16—C17—H17	119.8
C10—P2—Rh1	116.84 (6)	C18—C17—H17	119.8
C16—P2—Rh1	117.12 (6)	C19—C18—C17	120.11 (19)
C3—C2—C7	118.24 (19)	C19—C18—H18	119.9
C3—C2—P2	123.22 (15)	C17—C18—H18	119.9
C7—C2—P2	118.52 (16)	C20—C19—C18	119.90 (18)
C4—C3—C2	120.8 (2)	C20—C19—H19	120
C4—C3—H3	119.6	C18—C19—H19	120
C2—C3—H3	119.6	C19—C20—C21	120.27 (19)
C5—C4—C3	121.7 (2)	C19—C20—H20	119.9
C5—C4—H4	119.2	C21—C20—H20	119.9
C3—C4—H4	119.2	C20—C21—C16	120.26 (18)
C4—C5—C6	117.4 (2)	C20—C21—H21	119.9
C4—C5—C8B	140.7 (5)	C16—C21—H21	119.9
C6—C5—C8B	101.4 (5)	C9A—C8A—C5	122.6 (5)
C4—C5—C8A	113.1 (3)	C9A—C8A—H8A	118.7
C6—C5—C8A	129.4 (3)	C5—C8A—H8A	118.7
C7—C6—C5	121.6 (2)	C8A—C9A—H9A1	120
C7—C6—H6	119.2	C8A—C9A—H9A2	120
C5—C6—H6	119.2	H9A1—C9A—H9A2	120
C6—C7—C2	120.3 (2)	C8B—C9B—H9B1	120
C6—C7—H7	119.9	C8B—C9B—H9B2	120
C2—C7—H7	119.9	H9B1—C9B—H9B2	120
C15—C10—C11	118.91 (17)	C9B—C8B—C5	114.4 (8)
C15—C10—P2	118.66 (14)	C9B—C8B—H8B	122.8
C11—C10—P2	122.43 (15)	C5—C8B—H8B	122.8
			
C01^i^—Rh1—P2—C2	−126.99 (17)	Rh1—P2—C10—C15	59.78 (15)
C01—Rh1—P2—C2	53.01 (17)	C2—P2—C10—C11	1.31 (17)
Cl1^i^—Rh1—P2—C2	48.71 (7)	C16—P2—C10—C11	110.36 (15)
Cl1—Rh1—P2—C2	−131.29 (7)	Rh1—P2—C10—C11	−120.47 (14)
C01^i^—Rh1—P2—C10	−9.16 (17)	C15—C10—C11—C12	−1.2 (3)
C01—Rh1—P2—C10	170.84 (17)	P2—C10—C11—C12	179.06 (15)
Cl1^i^—Rh1—P2—C10	166.53 (7)	C10—C11—C12—C13	0.1 (3)
Cl1—Rh1—P2—C10	−13.47 (7)	C11—C12—C13—C14	1.0 (3)
C01^i^—Rh1—P2—C16	112.47 (17)	C12—C13—C14—C15	−1.1 (3)
C01—Rh1—P2—C16	−67.53 (17)	C13—C14—C15—C10	0.1 (3)
Cl1^i^—Rh1—P2—C16	−71.84 (7)	C11—C10—C15—C14	1.1 (3)
Cl1—Rh1—P2—C16	108.16 (7)	P2—C10—C15—C14	−179.15 (15)
C10—P2—C2—C3	99.98 (17)	C2—P2—C16—C17	−96.18 (16)
C16—P2—C2—C3	−6.79 (18)	C10—P2—C16—C17	156.22 (16)
Rh1—P2—C2—C3	−134.20 (15)	Rh1—P2—C16—C17	27.23 (17)
C10—P2—C2—C7	−78.41 (16)	C2—P2—C16—C21	83.97 (16)
C16—P2—C2—C7	174.82 (15)	C10—P2—C16—C21	−23.63 (17)
Rh1—P2—C2—C7	47.41 (16)	Rh1—P2—C16—C21	−152.63 (13)
C7—C2—C3—C4	1.0 (3)	C21—C16—C17—C18	−1.3 (3)
P2—C2—C3—C4	−177.38 (16)	P2—C16—C17—C18	178.86 (16)
C2—C3—C4—C5	1.1 (3)	C16—C17—C18—C19	1.3 (3)
C3—C4—C5—C6	−1.9 (3)	C17—C18—C19—C20	−0.3 (3)
C3—C4—C5—C8B	168.8 (5)	C18—C19—C20—C21	−0.9 (3)
C3—C4—C5—C8A	−180.0 (2)	C19—C20—C21—C16	0.9 (3)
C4—C5—C6—C7	0.6 (3)	C17—C16—C21—C20	0.2 (3)
C8B—C5—C6—C7	−173.4 (3)	P2—C16—C21—C20	−179.99 (15)
C8A—C5—C6—C7	178.3 (3)	C4—C5—C8A—C9A	−171.1 (3)
C5—C6—C7—C2	1.5 (3)	C6—C5—C8A—C9A	11.1 (5)
C3—C2—C7—C6	−2.3 (3)	C8B—C5—C8A—C9A	−5.9 (6)
P2—C2—C7—C6	176.19 (15)	C4—C5—C8B—C9B	6.1 (10)
C2—P2—C10—C15	−178.44 (14)	C6—C5—C8B—C9B	177.7 (6)
C16—P2—C10—C15	−69.39 (16)	C8A—C5—C8B—C9B	−15.6 (5)

Symmetry code: (i) −*x*+1, −*y*+1, −*z*+1.

### Hydrogen-bond geometry (Å, º)

**Table:**

*D*—H···*A*	*D*—H	H···*A*	*D*···*A*	*D*—H···*A*
C9*B*—H9*B*1···O01^ii^	0.93	2.54	3.205 (11)	129
C14—H14···Cl1^iii^	0.93	2.79	3.660 (3)	157

Symmetry codes: (ii) −*x*+2, −*y*, −*z*; (iii) −*x*+1, −*y*+2, −*z*+1.
